# Rubinstein-Taybi Syndrome Associated with Pituitary Macroadenoma: A Case Report

**DOI:** 10.7759/cureus.1151

**Published:** 2017-04-11

**Authors:** Yasamin Olyaei, J. Manuel Sarmiento, Serguei I Bannykh, Doniel Drazin, Robert T Naruse, Wesley King

**Affiliations:** 1 Department of Surgery, University of California, Riverside School of Medicine; 2 Department of Neurosurgery, Cedars-Sinai Medical Center; 3 Department of Pathology & Laboratory Medicine, Cedars-Sinai Medical Center; 4 Department of Anesthesiology, Cedars-Sinai Medical Center

**Keywords:** rubinstein-taybi syndrome, pituitary adenoma

## Abstract

Rubinstein-Taybi Syndrome (RSTS) is an autosomal dominant disorder that is classically characterized by prenatal and postnatal growth restriction, microcephaly, dysmorphic craniofacial features, broad thumbs and toes, and intellectual disability. We describe the first reported case of a pituitary macroadenoma associated with RSTS.

A 39-year-old Caucasian female with a past medical history of RSTS diagnosed at age two was found to have a gadolinium-enhancing pituitary mass on magnetic resonance imaging (MRI) of the brain three years ago during workup for migraine-like headaches. Subsequent serial imaging showed radiographic evidence of growth up to 11.5 x 14.0 x 10.0 mm in size. The pituitary sellar lesion was resected through an endoscopic transnasal transsphenoidal approach and was found to be a thyrotroph adenoma.

RSTS is a rare, neurodevelopmental genetic disease where most patients with disabilities survive into adulthood. The disorder is associated with an increased predisposition for development of nervous system tumors, including pituitary adenomas.

## Introduction

Rubinstein-Taybi syndrome (RSTS) is an autosomal dominant disorder characterized by mutations in the cyclic-AMP-regulated enhancer binding protein (CREBBP) gene in 1p13.3 and its homolog, E1A binding protein p300 (EP300) on chromosome 22. RSTS is clinically characterized by prenatal and postnatal growth restriction, microcephaly, dysmorphic craniofacial features, broad thumbs and toes, and intellectual disability. Other common associated findings include syndactyly, musculoskeletal anomalies including patellar dislocations, scoliosis and cervical spondylolisthesis, and congenital heart abnormalities such as patent ductus arteriosus, arterial septal defects and ventricular septal defects. Over 90% of RSTS individuals with disabilities survive to adulthood, and healthcare for these patients is complex, involving an interdisciplinary approach across multiple subspecialties [1].

Patients with RSTS have an increased predisposition for the development of tumors of the nervous system [2]. We describe the first reported case of a pituitary macroadenoma in a middle-aged female with RSTS that was successfully treated with an endoscopic transnasal transsphenoidal approach.

## Case presentation

### Presenting symptoms and workup

A 39-year-old Caucasian female, with a past medical history of RSTS diagnosed at age two, presented with new persistent migraine-like headaches of several weeks duration. A magnetic resonance imaging (MRI) of the brain performed in 2012 showed a 10 mm intrasellar, gadolinium-enhancing mass enlarging non-functional pituitary macroadenoma, which was followed for three years. Her past medical history is significant for anxiety, chronic nasal obstruction, congenital macroglossia, and chronic sinusitis. Her past surgical history is significant for repair of polydactyly at birth, mandibular surgery for significant prognathism and correction of nasal septum at 13 years of age, and surgical repair of large atrial septal defect at age 18. Medications include aspirin, escitalopram, lorazepam, and oxymetazoline. She has not had genetic testing for gene mutation analysis, and she is the middle child of a family with an elder female sister and younger male brother, neither of whom exhibit any characteristics of RSTS. On physical examination, she exhibited maxillofacial dysmorphism and some mild cognitive delay, but otherwise was neurologically intact with normal visual fields.

**Figure 1:**
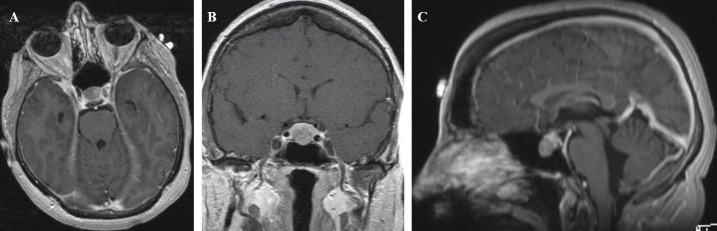
Preoperative T1-weighted MRI brain with and without gadolinium in the axial (A), coronal (B), and sagittal (C) planes showing a 11.5 x 14.0 x 10.0 mm hypoenhancing mass in the inferior pituitary gland causing upward bowing of the diaphragma sella and mild sellar expansion.

Follow-up neuroimaging in 2015 showed evidence of tumor growth and prompted surgical intervention. MRI brain with and without gadolinium revealed a 11.5 x 14.0 x 10.0 mm hypoenhancing mass in the inferior pituitary gland, causing upward bowing of the diaphragma sella and mild sellar expansion (Figure 1). There was no mass effect on the optic chiasm. Her preoperative hormone panel was normal except for elevated follicle stimulating hormone (FSH) at 25 mIU/ml, but this hormone level was drawn one week after the patient had restarted DepoProvera for irregular menstrual cycles.

### Surgical approach

The patient underwent an endoscopic transnasal transsphenoidal craniotomy for pituitary tumor excision. The patient has had extensive maxillofacial surgery due to her RSTS. She was also found to have a nasal septal perforation and severely deviated septum to the left. Therefore, an ear, nose, and throat (ENT) surgeon was required for the endoscopic approach to the sella turcica. Bilateral endoscopic sphenoidotomies and septoplasty were performed to expose the rostrum of the sphenoid sinus, which was removed to identify the sella and site of the tumor. A white-colored, fibrotic tumor was encountered and dissected from the adjacent pituitary tissue. Image guidance was then used for a volumetric excision. Multiple Valsalva maneuvers were performed without evidence of cerebrospinal fluid leak. The floor of the sella turcica was reconstructed using bone and septal cartilage from the exposure and layered with fibrin glue.

### Pathology

**Figure 2:**
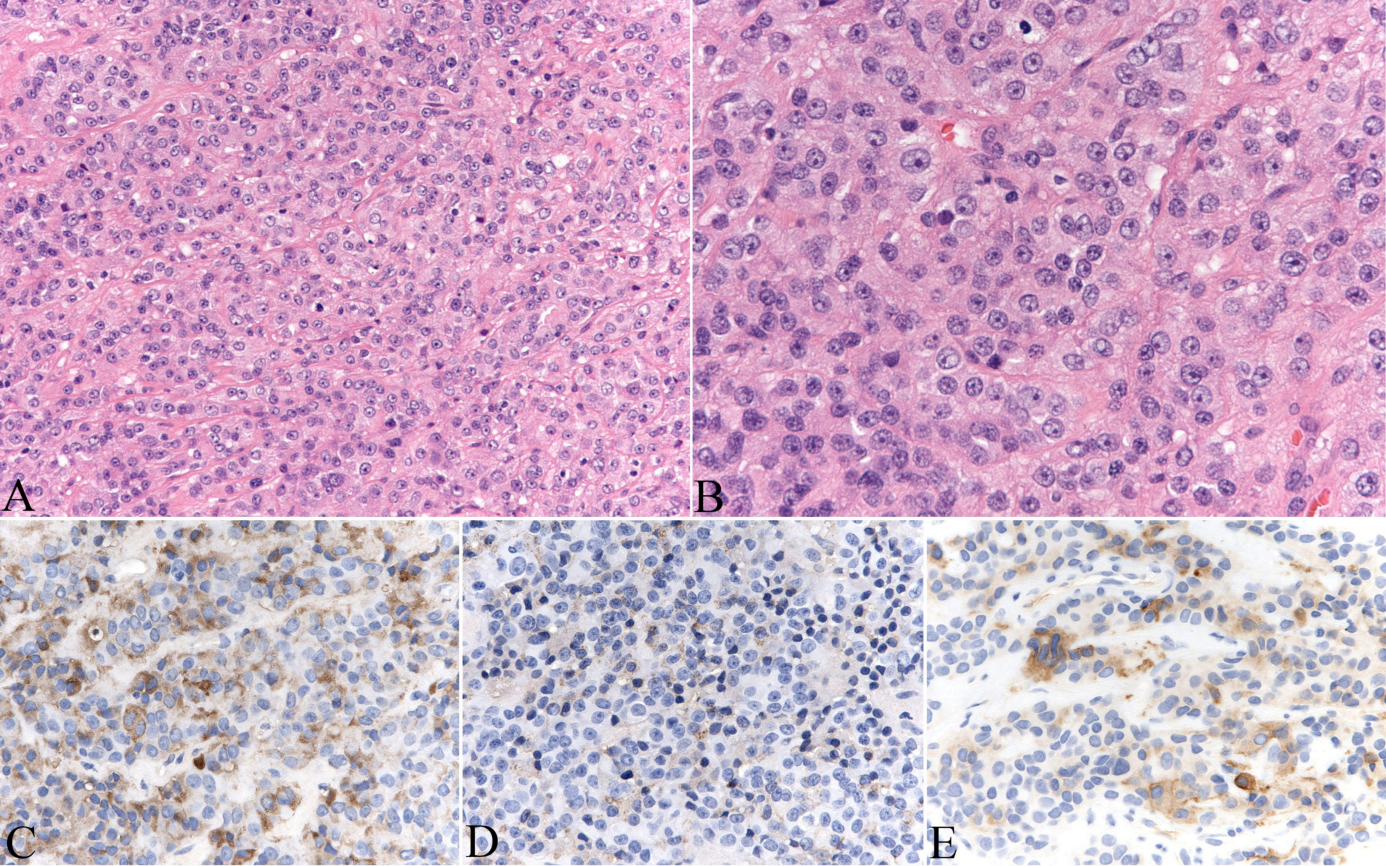
Hematoxylin and eosin stained sections of the pituitary lesions show neuroendoctine neoplasm (A, B). The tumor cells express TSH (C), growth hormone (D) and alpha subunit (E). Original magnifications: 200 x (A, C-E), 400 x (B). TSH - thyroid stimulating hormone

Excisional biopsy of the pituitary lesion showed expanded acini and trabeculae of minimally pleomorphic oval nuclei with conspicuous nucleoli associated with eosinophilic granular cytoplasm (Figure 2). Immunohistochemistry results showed positivity for thyroid stimulating hormone (TSH) in up to 60% of tumor cells. Additionally, up to two percent of tumor cells were weakly positive for growth hormone, and up to five percent of tumor cells were stained for alpha-subunit human chorionic gonadotropin (HCG). There was no staining for FSH, luteinizing hormone (LH), prolactin and adrenocorticotropic hormone (ACTH). No necrosis of appreciable mitotic activity was detected, and the ki-67 proliferative index was two percent. 

### Postoperative course

The patient was extubated following surgery but developed acute airway obstruction due her pre-existing macroglossia. She was reintubated to secure the airway and was monitored overnight in the intensive care unit, where she was successfully extubated the following day. A consideration to facilitate reintubation in this patient with a known difficult airway would be utilizing an airway exchange catheter as a bridge to a difficult extubation. She was placed on hydrocortisone taper and the remainder of her postoperative course was uneventful. She was discharged on postoperative day 2 without evidence of diabetes insipidus or cerebrospinal fluid leak.

## Discussion

This case describes an adult female with RSTS and clinically non-functional, TSH-positive pituitary macroadenoma. RSTS is a rare, neurodevelopmental and plurimalformative autosomal dominant genetic disease with a low population prevalence (one case per 125,000 live births). Although there are no precise and standard diagnostic criteria for RSTS, individuals have distinctive features including typical facial features, microcephaly, broad thumbs and first toes, intellectual disability and postnatal growth retardation. Other organs and systems may be affected as well; however, there is no particular sign or symptom that is pathognomonic for RSTS.

### Clinical features of RSTS

Facial features in RSTS are characterized by low frontal hairline, downslanting of palpebral fissures, dysplastic and low-set ears, an arched palate, mild micrognathia, and dental abnormalities [1]. Other known clinical malformations include optic nerve colobomas, talon cusps, congenital heart diseases, keloid formation, obesity, and obstructive sleep apnea. Congenital anomalies of cervical vertebrae, including os odontoideum, hypoplasia of the dens, and C1-C2 instability represent a major sequelae of RSTS [3-4]. This may result in stenosis at the craniovertebral junction leading to cervical myelopathy and predisposition to quadriparesis in the event of mild trauma. Other neurodevelopmental disorders associated with RSTS include dysgenesis of the corpus callosum [5-6], Dandy Walker malformation [7-8], Arnold Chiari malformation type 1 with and without syringomyelia [9-12], and tethered cord syndrome [13]. Dissecting aneurysms have also been associated with RSTS in the literature. Fischer, et al. reported a spontaneous extracranial internal carotid artery dissecting aneurysm and occlusion of both vertebral arteries in a 21-year-old female with RSTS that led to medullary infarct [14]. Ishizaka, et al. reported a cerebral infarction in a 44-year-old man with a dissecting aneurysm of the anterior cerebral artery [15].

### Nervous system tumors in Rubinstein-Taybi syndrome

There is a known pattern of increased predisposition for tumors of the brain and of tissue derived from neural crest origins in patients with RSTS. The estimated incidence of such types of tumors, malignant or benign, in patients with RSTS is five percent [2]. An epidemiological study by researchers from the Cincinnati Center for Developmental Disorders reported 36 tumors in a registry of 724 patients with RSTS from 1989 to 1994; of these 36, 12 (33%) were from neural origins [2]. There were six malignant tumors (one oligodendroglioma, one oligoastrocytoma, two medulloblastomas, and two neuroblastomas) and six benign tumors (two meningiomas, one pheochromocytoma, one neurilemmoma, one pineocytoma, and one pituitary microadenoma). Both meningiomas occurred in middle age (39 and 41 years). The pituitary microadenoma was reported through personal communication and was an incidental finding in a 49-year-old Caucasian male. Immunohistochemistry analysis showed growth hormone positivity only. More tumors of the central and peripheral nervous system have been reported over the subsequent years, furthering the positive correlation between RSTS and nervous system tumors [16-19]. This case report describes the first reported pituitary macroadenoma in a patient with RSTS. The immunoprofile was of thyrotroph adenoma with minimal co-expression of growth hormone and alpha subunit. The low percentage of cells with co-expression of growth hormone was insufficient to produce any serum elevation of growth hormone or clinical evidence of acromegaly. The intensity and density of TSH-positive cells in this tumor was relatively low, which may explain the silent clinical presentation and normal TSH levels.

### Genetics of Rubinstein-Taybi syndrome

A genetic cause of RSTS was first discovered in 1991, when a de novo reciprocal translocation with breakpoints in chromosomal region 16p13.3 was observed in some afflicted patients. Molecular analysis of 16p13.3 identified mutations in the CREBBP gene in approximately 50% of patients with RSTS [20-22]. Mutations in another important house-keeping gene, EP300 on chromosome 22, has been associated with RSTS in approximately five to eight percent of cases [21,23]. Despite our advances in genetic testing for diagnosing RSTS, many cases have no identifiable genetic cause and most cases are currently diagnosed based on clinical features [1]. Further research is needed to identify genotype-phenotype correlations and new candidate genes for future therapeutic targeting of epigenetic components altered in RSTS [24].

## Conclusions

RSTS is a rare, neurodevelopmental autosomal dominant genetic disease where most patients with disabilities survive into adulthood. The disorder is associated with an increased predisposition for development of nervous system tumors, including pituitary adenomas. We now add a pituitary macroadenoma to the literature regarding tumors associated with RSTS.
